# Quantitative Effect of Suboptimal Codon Usage on Translational Efficiency of mRNA Encoding HIV-1 *gag* in Intact T Cells

**DOI:** 10.1371/journal.pone.0002356

**Published:** 2008-06-04

**Authors:** Kholiswa C. Ngumbela, Kieran P. Ryan, Rohini Sivamurthy, Mark A. Brockman, Rajesh T. Gandhi, Nina Bhardwaj, Daniel G. Kavanagh

**Affiliations:** 1 HIV Pathogenesis Programme, Doris Duke Medical Research Institute, University of KwaZulu Natal, Durban, South Africa; 2 Partners AIDS Research Center, Massachusetts General Hospital and Division of AIDS, Harvard Medical School, Boston, Massachusetts, United States of America; 3 New York University School of Medicine, New York, New York, United States of America; University of California San Francisco, United States of America

## Abstract

**Background:**

The sequences of wild-isolate strains of Human Immunodeficiency Virus-1 (HIV-1) are characterized by low GC content and suboptimal codon usage. Codon optimization of DNA vectors can enhance protein expression both by enhancing translational efficiency, and by altering RNA stability and export. Although *gag* codon optimization is widely used in DNA vectors and experimental vaccines, the actual effect of altered codon usage on *gag* translational efficiency has not been quantified.

**Methodology and Principal Findings:**

To quantify translational efficiency of *gag* mRNA in live T cells, we transfected Jurkat cells with increasing doses of capped, polyadenylated synthetic mRNA corresponding to wildtype or codon-optimized *gag* sequences, measured Gag production by quantitative ELISA and flow cytometry, and estimated the translational efficiency of each transcript as pg of Gag antigen produced per µg of input mRNA. We found that codon optimization yielded a small increase in *gag* translational efficiency (approximately 1.6 fold). In contrast when cells were transfected with DNA vectors requiring nuclear transcription and processing of *gag* mRNA, codon optimization resulted in a very large enhancement of Gag production.

**Conclusions:**

We conclude that suboptimal codon usage by HIV-1 results in only a slight loss of *gag* translational efficiency per se, with the vast majority of enhancement in protein expression from DNA vectors due to altered processing and export of nuclear RNA.

## Introduction

HIV gene expression is controlled by multiple complex regulatory mechanisms [1], [2]. HIV structural proteins are expressed from unspliced 9 kb (*gag/pol*) and partially-spliced 4 kb (*env*) transcripts that are unstable and that cannot be efficiently exported from the nucleus in the absence of the HIV regulatory protein Rev. The lack of nuclear stability and export in the absence of Rev is partly due to the presence of defined inhibitory sequences (known as INS, IN, and CRS) within the structural genes themselves. In addition, the low GC content of the HIV RNA also contributes to nuclear instability, even in the absence of defined inhibitory sequences [3]. Rev rescues 9 kb and 4 kb HIV RNA from nuclear degradation by binding to the Rev Response Element (RRE) located within the Env region of the genome, stabilizing the RNA, and promoting RNA export [4].

For HIV-uninfected cells that are transfected with *gag* DNA, Gag expression is greatly enhanced by codon optimization [5]-[11]. Codon optimization originated as a means to enhance the translational efficiency of genes with codon usage that was sub-optimal for the transfected cell type. Increasing evidence suggests that the enhancement of Gag expression by codon optimization in DNA-transfected cells is largely attributable to changes in nuclear RNA stability and export, rather than to enhanced translational efficiency [10], [12]-[14]. For example, in contrast to DNA transfection, when non-optimized genes were expressed from cytoplasmic Sindbis vectors [10] or synthetic mRNA [14], production of Gag and Env proteins was readily detectable by Western blot and ELISA. These results indicate that access to the cytoplasm is a major limiting factor for expression of non-codon-optimized sequences. However previous studies have not made precise comparisons of the translational efficiency of codon-optimized versus non-optimized *gag* RNA, and it is not known to what degree codon usage *per se* affects translational efficiency of HIV transcripts.

To compare the translational efficiency of different transcripts in the cytoplasm of transfected cells for the present study, we generated synthetic mRNA encoding codon-optimized or non-optimized *gag* sequences, and transfected Jurkat cells by electroporation in the presence of increasing amounts of synthetic mRNA. This method introduces mRNA directly to the cytoplasm, thus bypassing the requirement for mRNA transcription, processing and nuclear export. We have previously shown that over 95% of cells subjected to electroporation under similar conditions express the mRNA-encoded product [15]. By measuring the amount of Gag produced per pmol of mRNA, we estimated the translational efficiency of each transcript. For comparison, we transfected Jurkat cells with DNA vectors encoding the corresponding open reading frames for codon-optimized or non-optimized gag under the control of the CMV-IE promoter. Our results demonstrate that the vast majority of enhancement of protein expression achieved by codon optimization of DNA vectors is unrelated to codon usage per se, and actually mediated by pre-translational effects, such as improved mRNA stability and transport.

## Results

**Figure 1:**
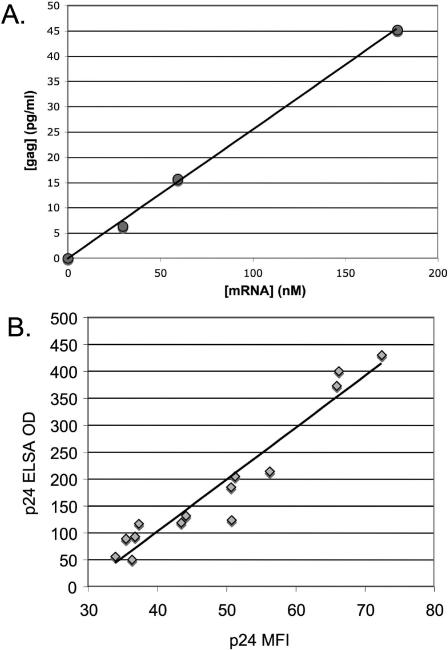
Gag expression in Jurkat cells transfected with *gag* mRNA. A. Gag production correlates with mRNA concentration. Jurkat cells were transfected by electroporation with increasing doses of synthetic codon-optimized gag mRNA. After 24 hours, the amount of Gag in the supernatant was determined by ELISA. The line shows linear fit (R^2^>0.99). B. The amount of Gag in cell supernatants is representative of the median Gag production in electroporated cells. Jurkat cells were transfected with varying doses of gag mRNA; after 24 hours, cell supernatants were harvested for ELISA, and cell pellets were fixed, permeabilized, and stained with anti-Gag/p24 antibody. Gag production as measured by ELISA is shown on the Y axis; Gag production as measured by MFI (median fluorescence intensity) is shown on the X axis. Line shows linear fit (R^2^ = 0.93). ELISA values are the average of triplicate wells.

To measure Gag production by transfected cells, we generated synthetic mRNA encoding HIV-1 *gag*. Capped, polyadenylated mRNA was transfected into Jurkat cells by electroporation. Gag protein production was quantified by measuring the concentration of Gag p24 in cell supernatant by ELISA and by staining transfected cells with Gag-p24 specific fluorescent antibody. We found that the amount of Gag released into the culture medium was proportional to the amount of mRNA added to the transfection reaction (Fig 1A, R ^2^>0.99). The amount of Gag detected by ELISA was proportional to the median fluorescence intensity (MFI) of cells stained with p24-specific fluorescent antibody (Figure 1B, R ^2^ = 0.93), demonstrating that the amount of secreted Gag detected in cell supernatants was indicative of protein production by the majority of cells in the population.

**Figure 2:**
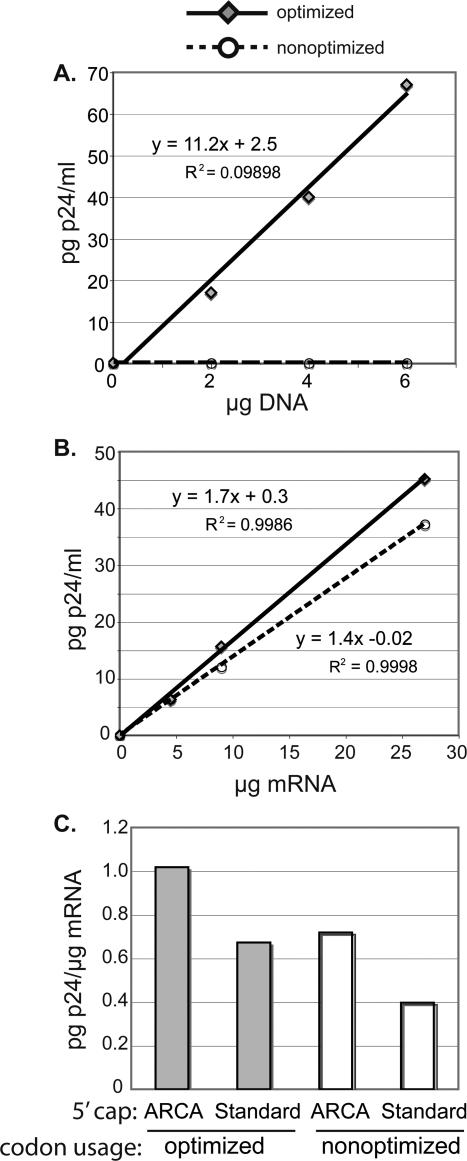
Relative efficiency of Gag production from codon-optimized and non-optimized sequences. A. Jurkat cells were transfected with increasing amounts of plasmid DNA encoding codon-optimized or non-optimized *gag* under control of the CMV-IE promoter, and Gag production was measured by ELISA analysis of culture supernatants. For codon-optimized sequence, Gag production was directly proportional to the amount of input DNA. In contrast, for non-optimized sequence no significant Gag production was detectable at any dose of DNA. Representative of two independent experiments. B. In order to measure translational efficiency of mRNA introduced directly into the cytoplasm, cells were transfected with increasing doses of synthetic mRNA transcribed from codon-optimized or non-optimized templates. The template sequences of the open reading frames were identical to those used for the DNA transfections shown in A above. To estimate the translational efficiency (amount of Gag produced per input µg mRNA), we calculated the slope of the curve as shown. Representative of four independent experiments. C. To determine the effects of modified 5′ cap on translational efficiency, cells were transfected with increasing amounts of codon-optimized or non-optimized mRNA generated with conventional m^7^G(5′)ppp(5′)G cap analog, or with anti-reverse cap analog (ARCA; which has been previously reported to enhance translational efficiency of synthetic mRNA). The Y axis shows the translational efficiency the mRNA transcripts, derived from the slope of the curves as shown for B above.

To compare the translational efficiency of different transcripts, we used codon optimized or non-optimized *gag* sequences derived from an HIV-1 Clade C clinical isolate[8]. To confirm that Gag was poorly expressed from non-optimized transcripts transcribed in the nucleus, we transfected Jurkat cells with DNA expression vectors encoding optimized or nonoptimized *gag* sequences under control of the CMV promoter. For optimized vector, we used the previously described plasmid p96ZM651gag-opt[8]; for non-optimized vector, we constructed plasmid p96ZM651gag-NonOpt2, encoding the matched non-optimized wildtype open reading frame. Sequence analysis confirmed that the two vectors were identical with respect to both the predicted peptide sequence of the translational product and the 5′ and 3′ untranslated regions, and differed only with respect to codon usage within the open reading frame. As shown in Figure 2A, p24 was easily detectable in the supernatant of cells transfected with codon-optimized DNA vector, and was directly proportional to the concentration of plasmid in the transfection. In contrast, no significant Gag production was detected in cells transfected with the wild-type sequence, consistent with the severe defect of protein expression previously reported for non-optimzed sequence [8].

To measure the actual translational efficiency of optimized and nonoptimized mRNA delivered directly to the cytoplasm, we constructed transcriptional template vectors encoding identical open reading frames as p96ZM651gag-opt or p96ZM651gag-NonOpt2 above, and performed *in vitro* transcription to generate capped, polyadenylated synthetic mRNA. Jurkat cells were transfected with increasing amounts of mRNA, and the amount of Gag in the supernatant after 24 hours was measured by ELISA. The experiment was repeated for four separate batches of synthetic mRNA, and the relative translational efficiency for each transcript was estimated by calculating the slope of the curve (pg p24/ µg mRNA). Figure 2B shows dose-dependent Gag production for one representative of four independent experiments. The translational efficiency of the optimized sequence was only slightly enhanced compared to the non-optimized sequence (1.7 pg vs 1.4 pg p24/ µg mRNA respectively). Among the four independent experiments the ratio of the average translational efficiency of optimized to non-optimized mRNA ranged from 1.2 to 1.9 (not shown), with an mean ratio of 1.6. Thus codon optimization resulted in an approximately 60% increase in translational efficiency.

To validate our comparison of translational efficiencies we compared the effect of codon optimization to the effect of another method of enhancing translational efficiency. For experiments described above transfections were carried out using mRNA capped with conventional m^7^G(5′)ppp(5′)G cap analog. Translational efficiency of synthetic mRNA can be enhanced by the use of an anti-reverse cap analog (ARCA) [16], [17]. We compared the translational efficiency of codon-optimized and non-optimized mRNA synthesized with ARCA or conventional m^7^G(5′)ppp(5′)G cap analogs. Figure 2C shows that, consistent with results shown in 2B, when Gag production was measured 22 hours after transfection, the effect of codon optimization for all transcripts was an enhancement of between 1.4 fold and 1.7 fold. Figure 2C also shows that use of the ARCA cap enhanced translation by between 1.5 and 1.8 fold. This enhancement is comparable to the 2.3-2.6 fold enhancement in luciferase production reported for use of ARCA in a reticulocyte lysate translation system [17]. Figure 2C also shows that translational enhancement by codon optimization and by use of ARCA cap is additive.

## Discussion

Constructs bearing codon-optimized genes are widely used to enhance heterologous expression of lentiviral gene products. However, the term “codon optmization” is potentially confusing, in that it implies that enhancement of expression is directly related to codon usage and to translational efficiency. Here we report the first quantitative assessment of the effect of codon optimization on HIV-1 *gag* translational efficiency. Our results are consistent with the conclusions of previous reports that for DNA-transfected cells, the majority of enhancement in protein expression by *gag* codon optimization is in fact due to increased stability and export of nuclear mRNA. Nevertheless, we find a reproducible enhancement (in four out of four experiments) of Gag production of approximately 60% that can be attributed to codon optimization. We propose that this value represents the actual difference in translational efficiency due to suboptimal codon usage by clinical-isolate HIV-1, although we also cannot rule out a possible contribution of altered cytoplasmic mRNA stability based on available data. Why a virus that has evolved to replicate in human cells would maintain suboptimal codon usage is unexplained. It is worth noting that the cellular editing enzyme APOBEC3G has a cytidine deaminase activity that can induce G»A hypermutation in the sequence of replicating HIV [18]-[20]; thus there may be evolutionary pressure for HIV to use the minimal number of G-containing codons as possible, even if it results in a slight reduction of translational efficiency.

Finally we note that the combined effects of codon optimizaton and ARCA cap (shown in Figures 2B and 2C) yielded a total increase in protein production of approximately 2.3 fold compared to wildtype transcript sequence with a conventional 5′ cap. While small compared to the effects of codon optimization for DNA transfection, this enhancement may still be useful in light of increasing applications of RNA transfection for vaccine development [14], [15], [21], [22].

## Materials and Methods

### Vectors

The non-optimized and optimized *gag* sequences were derived from plasmids p96ZM651.8 (GenBank Accession #AF286224), which contains the near full length molecular clone of an HIV-1 subtype C clinical isolate from Zambia; and p96ZM651gag-opt (GenBank accession #AY181195), which contains a codon-optimized *gag* sequence of p96ZM651 cloned into EcoRI and BamHI cloning sites, 5′→3′ of vector pcDNA3.1(-). p96ZM651gag-opt and p96ZM651.8 were provided by the AIDS Research and Reference Reagent Program, Division of AIDS, NIAID, NIH (from Drs. Yingying Li, Feng Gao, and Beatrice H. Hahn [8]). For DNA transfection with optimized sequence Jurkats were transfected with p96ZM651gag-opt. For DNA transfection with nonoptimized sequence we constructed a new plasmid (p96ZM651gag-NonOpt2). In order to construct p96ZM651gag-NonOpt2, we sequenced p96ZM651gag-opt and determined that the 5′ upstream sequence including the vector EcoRI site and the start codon was 5′GAATTCGGAAGACCCAGGAGAGAGATG, and the 3′ downstream sequence including the stop codon and the vector BamHI site was 5′TAATGAGACGTGAGGATCC. Using these sequences we designed specific PCR primers to clone the nonoptimized *gag* from p96ZM651.8 into the EcoRI and BamHI cloning sites of p96ZM651gag-opt, yielding a vector (p96ZM651gag-NonOpt2) with identical 5′ and 3′ untranslated regions to p96ZM651gag-opt.

Templates for *in vitro* transcription (IVT) of gag mRNA were constructed by cloning the codon-optimized and non-optmized *gag* sequence into template vectors consisting of: T7 polymerase promoter, Kozak ribososme binding site, *gag* open reading frame, 64 nt poly-adenosine tail, AscI restriction site for linearization. All plasmids were sequenced before use to confirm correct sequences.

### Transfections

Synthetic mRNA was transcribed from the template plasmids using Message Machine T7 or (T7 Ultra kit for ARCA-capped mRNA) (Ambion). mRNA was purified with RNEasy kit (Qiagen) and mRNA concentration was quantified with serial dilutions of mRNA solutions using a ND-1000 spectrophotometer (Nanodrop). For each transfection, 10^6^ cells were washed twice with OptiMem (Gibco) and resuspended in 300 µl of OptiMem with the indicated concentration of mRNA or plasmid DNA, left on ice for five minutes, and transfected by electroporation with a GenePulserII (Biorad) at 900V/0.75mSec in a 4 mm cuvette. Cells were then cultured in RPMI plus 10% fetal calf serum for 24 hours, after which conditioned medium was harvested for analysis by p24 ELISA kit (PerkinElmer).

### p24 determination

Transfected cells were cultured in RPMI plus 10% fetal calf serum for 24 hours (RNA transfection) or 48 hours (DNA transfection), after which conditioned medium was harvested for analysis by p24 ELISA kit (PerkinElmer). Cell pellets were fixed and permeablized using Fix/Perm kit (Becton Dickinson, San Jose, CA), stained with anti-p24 antibody (KC-57-PE Beckman Coulter), and analyzed on a BD FacsCalibur flow cytometer. MFI was determined by gating on all live cells.
